# PulseSelect vs FARAPULSE pulsed field ablation: Comparative analysis of myocardial, neural-injury and hemolysis biomarkers and short-term outcomes

**DOI:** 10.1016/j.ijcha.2026.101957

**Published:** 2026-06-18

**Authors:** Christian Gold, Anastasia Falagkari, Florian Post, Victoria Johnson, Esther Roth, Sinan Justin Kühn, Dominik Linz, Dobromir Dobrev, Julia Erath-Honold, Laura Rottner, David M. Leistner, Jana Kupusovic, Reza Wakili

**Affiliations:** a Department of Cardiology and Angiology, University Hospital Frankfurt, Theodor-Stern-Kai 7, 60590 Frankfurt am Main, Germany; b German Centre for Cardiovascular Research (DZHK), partner site Rhine-Main, Germany; c Department of Cardiology, Maastricht University Medical Center and Cardiovascular Research Institute Maastricht, Maastricht, the Netherlands; d Department of Biomedical Sciences, Faculty of Health and Medical Sciences, University of Copenhagen, Copenhagen, Denmark; e University of Duisburg-Essen Institute of Pharmacology, Essen, Germany; f Department of Medicine and Research Center, Montreal Heart Institute and Université de Montréal, Montreal, Canada; g Department of Integrative Physiology, Baylor College of Medicine, Houston, TX, USA

**Keywords:** PFA, Atrial fibrillation, Pulmonary vein isolation, Myocardial biomarkers, S100, Hemolysis

## Abstract

**Background:**

Randomized trials have demonstrated safety and efficacy of pulsed field ablation (PFA) for atrial fibrillation (AF) using the FARAPULSE™ system (PFA—F). We evaluated implementation of the PulseSelect™ system (PFA—P) in a PFA-F experienced center.

**Methods:**

This single-center study included 150 consecutive patients undergoing first pulmonary vein isolation (PVI) with PFA-P (*n* = 75) or PFA-F (n = 75). Procedural characteristics, acute efficacy, safety endpoints, AF recurrence rate, myocardial, neural and hemolysis biomarkers (high-sensitive troponin T, creatine kinase (CK), creatine kinase MB, bilirubin, haptoglobin, lactate dehydrogenase and S100) post ablation were analyzed and compared.

**Results:**

Baseline characteristics were comparable, except for a higher proportion of males in the PFA-F group. Acute PVI was comparable between groups (98% vs. 100%). Skin-to-skin time and radiation dose were similar, while fluoroscopy (14.3 vs. 11.9 min, *p* = 0.009) and LA-dwell time (18 vs. 14 min, *p* < 0.001) were higher in PFA—P. No major complication occurred, while minor complication rates were comparable. Cardiac biomarker release was significantly higher in PFA-P (ΔCK: 203 vs. 127 U/l**,***p* = 0.005; Δtroponin: 1212 vs. 1014 pg/ml, *p* = 0.035 (day 1)), whereas neural injury markers were similar. Hemolysis markers indicated greater changes with PFA-F (Δbilirubin: 0.3 vs. 0.2 mg/dl, *p* = 0.004; Δhaptoglobin: −46.5 vs. −27.5 mg/dl, *p* = 0.001 (day1)). Procedural parameters improved with increasing PFA-P experience.

**Conclusion:**

Implementation of PFA-P is feasible with an evident learning curve in a PFA-F experienced center. Higher post-procedural cardiac biomarker levels may suggest a greater extent of local myocardial tissue damage after PFA—P. The impact of biomarkers on long-term outcome requires further studies.

## Highlights

• Increased levels of CK and troponin in patients treated with PFA-P compared to PFA-F suggest higher extent of myocardial injury in patients undergoing PVI with PFA-P.
• Comparably low levels of the neural injury biomarker S100 after both PFA-P and PFA-F suggest no relevant bystander injury to intrinsic cardiac autonomic nervous system.
• Increased levels of bilirubin and decreased haptoglobin in PFA-F compared to PFA-P point to a potentially more pronounced hemolysis in patients treated with PFA-F.
• A significant learning curve for PFA-P in experienced single-shot-operators, reflected in reduction of skin-to-skin time, fluoroscopy time and radiation dosage in PFA-P observed after less than 25 ablations.
• Experienced single shot operators opt for direct transseptal puncture with the dedicated PFA-P sheath, eliminating the need for transseptal sheath exchange.

## 1 Introduction

Pulmonary vein isolation (PVI) has emerged as a cornerstone therapy for rhythm control in atrial fibrillation (AF) [1] with randomized controlled trials showing consistent superiority in restoring and maintaining sinus rhythm and cost effectiveness of PVI over antiarrhythmic drug therapy [2], [3], [4], [5].

Non-thermal pulsed field ablation (PFA) is an emerging technology, which uses high-voltage current in microsecond long trains to create electrical fields, in order to cause irreversible cell death through electroporation and destabilization of cell membranes [6]. PFA has been shown to be non-inferior to thermal ablation methods regarding freedom from AF and procedure related serious adverse events [7]. Because of its tissue sensitivity, PFA has a high safety profile [8].

The FARAPULSE™ Boston Scientific (PFA—F) catheter was the first developed PFA catheter for use in clinical practice [9]. In 2023 the PulseSelect™ Medtronic (PFA—P) catheter received FDA approval for the treatment of paroxysmal and persistent AF and was shown to be an effective and safe PVI technology [10]. However, PFA-P is yet to be compared in randomized controlled manner against other modalities and real-world comparative data between PFA-P and PFA-F ablation systems remain limited [11], [12].

The aims of this study was to compare procedural aspects, efficacy, safety and short-term outcome 6 months after PFA-PVI with two PFA single shot catheter systems, to compare dynamics of biomarkers of myocardial, neural injury and hemolysis parameters after ablation, and to assess a potential learning curve for PFA-P in a PFA-F experienced center.

## 2 Methods

### 2.1 Study design and population

This single center, prospective observational study included consecutive patients, who underwent first PVI for paroxysmal or persistent AF at a tertiary care academic center between July 2024 and September 2025. Adult patients with a history of paroxysmal or persistent AF and indication for catheter ablation, in accordance with AF guidelines [2] were eligible for the study. Exclusion criteria were prior ablation of the left atrium (LA), LA thrombus, contraindication for oral anticoagulation and other lesions besides PVI.

Clinical and procedural data were collected prospectively in an electronic database dedicated to clinical surveys. Data regarding past medical history was retrieved from the individual health records.

All patients provided written informed consent to the ablation procedures and anonymized data collection for research purposes. The study received institutional review board approval (Frankfurt arrhythmia registry: “FRAME” 2023–1379).

### 2.2 Ablation strategies and periprocedural management

The ablation procedures were performed according to our center's standard of care, which is in line with the 2024 expert consensus statement on catheter and surgical ablation of AF of the European Heart Rhythm Association [13]. Prior to ablation, patients underwent transesophageal echocardiography to rule out intracardiac thrombus formation. Direct oral anticoagulants were continued until the evening before the ablation and were resumed on the procedure day, 2-4 h after removal of the 6 h compression bandage, provided the vascular access sites appeared unremarkable upon clinical assessment. For patients under Vitamin K antagonists an INR range of 2–3, without interruption and without bridging was strived for. All patients with a CHA_2_DS_2_-VA-Score of ≥1 were advised to resume the oral anticoagulation permanently.

Ablations were performed in a high-volume PVI center (with >340 PVIs annually) by three experienced operators. At the time of the introduction of the PFA-P system all operators were familiar with the use of the PFA-F system with a minimum of 200 cases each.

PVIs were performed under deep conscious sedation by use of intravenous propofol and fentanyl under continuous hemodynamic monitoring. Dosage was adapted to the individual hemodanymic and respiration status as well as to the subjective pain assessment of the operator.

Catheter access was performed by 7 French (F) and 8F in case of PFA-P or 7F and 11F in the cases of PFA-F that were inserted in the right femoral vein using anatomical landmarks. A decapolar diagnostic catheter was placed in the coronary sinus. A 6F terumo™ sheath over the radial artery for blood pressure measurement and as access option for fluoroscopic landmarking the aortic root guiding transeptal puncture (TSP).

The ablation procedure was performed in compliance with the manufacturer's instructions with respect to flushing, insertion and shaping of the sheaths and catheters. The first 2 PVIs of each operator in the PFA-P group, were accompanied by a Medtronic clinical support expert on site.

TSP was performed with a Swartz Braided SL0/SL1 sheath (Abbott) depending on the operators` preference and a BRK™ Transseptal Needle, XS Series (Abbott) needle. Thereafter, the initial sheath was exchanged over a super-stiff™ wire for the steerable 13F FARADRIVE™ (Boston scientific) or the 10F FlexCath Contour™ (Medtronic) sheath and unfractionated heparin was administrated with a target activated clotting time (ACT) of 300–350 s. In 34 of the PFA-P procedures direct TSP via FlexCath Contour™ was performed. Following TSP, a selective pulmonary vein (PV) angiography took place using a 5F Multipurpose™ catheter. All procedures were performed solely under fluoroscopic guidance.

The Farawave™ ablation catheter was placed and navigated accordingly to the depicted PVs over an extra-stiff™ j-shaped guidewire. A set of 4 pulses in “basket” and 4 in “flower” catheter formation was delivered per vein with a voltage output of 2000 V as a biphasic waveform of 5 trains per application (2.5 s ablation for every application) in unsynchronized fashion. The catheter was rotated after every set of 2 applications in an overlapping manner. Additional energy trains were applied if no entrance block was observed.

PulseSelect™ circular ablation catheter was navigated to the PV ostia with help of the PV tracker guidewire Medtronic. Once in place, synchronized biphasic energy of 1500 V was delivered in 4-trains-pulses with a total number of 4 applications in the PV ostium and 4 at a more antral position. Between every other energy delivery, the catheter was rotated clockwise by 90° to cover up the designed catheter gap. Before performing ablation of the right PVs, a test-pulse of 40 V was given and the ablation was resumed in absence of phrenic nerve palsy. Bolus energy applications were administered in a case of residual conduction of the PVs until achieving an entrance block.

Atropine (1 mg i.v.) was administered in all patients before energy deliveries. No additional lesions besides the PVs were applied. No esophageal temperature monitoring was used in either group. At the end of the procedure hemostasis was achieved using a combination of a figure-of-8 suturing technique and a compression bandage, which was applied for 6 h.

All patients received post ablation transthoracic echocardiography to rule out pericardial effusion, an electrocardiogram (ECG) and clinical exam on day 1 and 2 to rule out early arrhythmia recurrence.

### 2.3 Blood collection and serum biomarkers

Blood collection was performed at 3 timepoints: 1) up to 24 h before the procedure; 2) day 1 (20–24 h) after the procedure; 3) 2 days (44 h–48 h) post ablation. Special care was taken to avoid artificial hemolysis. Cardiac biomarkers (high-sensitive troponin T, creatine kinase (CK), creatine kinase MB (CK-MB)), hemolysis markers (bilirubin, haptoglobin, lactate dehydrogenase, LDH) and S100 (a neural injury biomarker, released by the intrinsic cardiac autonomic nervous system, located near the outflow tract and the epicardial fat close to the PVs [14]) were analyzed.

The increase or decrease (Δ) of analyzed biomarkers was evaluated as follows:

Δ Biomarker day 1 to baseline = serum levels on day 1 post PVI - baseline.

Δ Biomarker day 2 to baseline = serum levels on day 2 post PVI - baseline.

### 2.4 Study outcomes and follow-up

Primary efficacy endpoints combined acute procedural success, defined as successful isolation of all PVs confirmed by visible entrance block and 6-month AT/AF recurrence rate, defined as documented AT/AF through a wearable 1‑lead ECG or 12‑lead ECG after an eight-week blanking period.

Primary procedural endpoints included skin-to-skin time (min), LA-dwell time (min), fluoroscopy time (min), radiation dose (Gy*cm^2^), administered dosage of fentanyl (mg) and propofol (mg).

Safety endpoints consisted of major (death, stroke, non-fatal myocardial infarction, pericardial tamponade requiring drainage, vascular major complication requiring intervention, atrioesophageal fistula, PV stenosis and persistent phrenic paralysis) and minor complications (vascular events resolved conservatively, pericardial effusion with no need for intervention, transient phrenic paralysis, aspiration).

### 2.5 Statistical analysis

Categorical variables are presented as numbers and percentages and continuous variables are summarized as mean and standard deviation (SD) or median and interquartile range (IQR) depending on normality of distribution. For continuous variables, normal distribution was assessed using the Shapiro-Wilk test, and the equality of variances was evaluated with Levene's test. When both assumptions—normal distribution and homogeneity of variances—were fulfilled, a two-sided independent samples *t*-test was used to compare means and when the assumption of equal variances was violated, Welch's test was applied. In cases where normal distribution was not upheld, the Mann-Whitney *U* test was used. The analysis of paired samples was performed either with a paired *t*-test, if the data were normally distributed or with the Wilcoxon - Rang test if the data were not normally distributed. Association between categorical variables was assessed using the Chi-square test, or the Fisher's exact test, when appropriate. *P*-values <0.05 were considered statistically significant. Survival analysis was performed using Kaplan-Meier analysis and survival curves were compared using the log-rank test and Cox regression analysis. All analyses were performed with GraphPad Prism (Version 10.4.1).

## 3 Results

### 3.1 Patient population

Baseline characteristics are depicted in Table 1. The study included 150 patients (PFA—P: *n* = 75, PFA—F: n = 75). Both groups consisted of predominantly male patients (PFA—P: 53% and PFA—F: 72%, *p* = 0.018) with a median age of 70 (PFA—P) and 69 (PFA—F) years. The incidence of paroxysmal AF was similar in both groups (55% vs. 57%, for PFA-P and PFA—F, respectively, *p* = 0.742).

**Table 1:** Baseline characteristics.

Variable	PFA-P (n = 75)	PFA-F (n = 75)	p-value
Male gender, n (%)	40 (53.3)	54 (72)	**0.018**
Age, median (IQR), years	70 (15)	69 (17.5)	0.774
BMI, median (IQR), kg/m^2^	26.3 (6.2)	26.7 (6.1)	0.934
Arterial hypertension, n (%)	50 (66.7)	54 (72)	0.479
Diabetes mellitus, n (%)	11 (14.7)	11 (14.7)	1.000
CKD, n (%)	8 (10.7)	7 (9.3)	0.785
eGFR, median (IQR), ml/min/1.73 m^2^	70.8 (33.2)	76.6 (34.8)	0.313
CAD, n (%)	17 (22.7)	18 (24)	0.847
ICM, n (%)	9 (12)	6 (8)	0.414
NICM, n (%)	9 (12)	4 (5.3)	0.245
CHA_2_DS_2_-VA Score, median (IQR)	3 (3)	2 (3)	0.747
LVEF, median (IQR), %	55 (15)	55 (15)	0.822
NT-proBNP, median (IQR), pg/mL	609 (835)	414 (690)	0.109
LA area, median (IQR), cm^2^	25 (6.0)	24 (7.4)	0.865
Paroxysmal AF, n (%)	41 (54.7)	43 (57.3)	0.742
AAD at baseline, n (%)	10 (13.3)	12 (16)	0.644
Time from AF diagnosis to PVI, median (IQR), days	193 (872)	234 (1438)	0.706

PFA-P, Pulseselect™; PFA—F, Farapulse™; BMI, Body-Mass- Index; CKD, chronic kidney disease; eGFR, estimated glomerular filtration rate; CAD, coronary artery disease; ICM, ischemic cardiomyopathy; NICM, non-ischemic cardiomyopathy; LVEF, left ventricular ejection fraction; NT-proBNP, N-terminal brain natriuretic peptide; LA, left atrium; AF, atrial fibrillation; AAD, antiarrhythmic drugs; PVI, pulmonary vein isolation; IQR, interquartile range.

### 3.2 Acute efficacy and short-term outcome

Acute procedural success with complete PVI was equally high in both groups (PFA—P: 99% vs. PFA—F: 100%) and was achieved with manufacturer recommended 8 impulses per vein in 82.7% in the PFA-P group and 77.3% in the PFA-F group (*p* = 0.501). There was no significant difference regarding mean number of applied impulses in total (PFA—P: 32.9 ± 2.50 vs. PFA—F: 32.6 ± 2.79, *p* = 0.461) or per specific PVs between the devices (**Suppl. Fig. 1**).

**Fig. 1:**
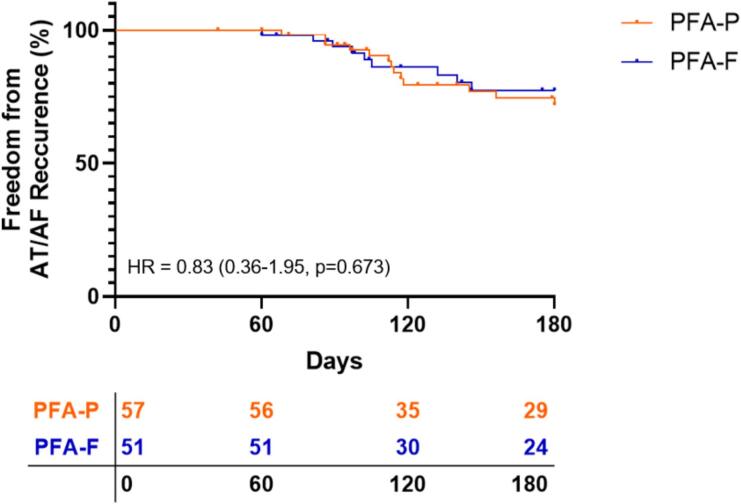
Freedom from AT/AF recurrence after 6 months following PVI with PFA-P and PFA-F. Kaplan–Meier curves showing comparable AT/AF recurrence rates at 6-month follow-up after an 8-week blanking period for PFA-P and PFA-F.

Median follow up time was 177 [77.3] days and follow up data were available for 57/75 patients in the PFA-P group and 51/75 PFA-F patients (*p* = 0.568). At 6-month follow-up, after an 8-week blanking period, AT/AF recurrence rates were comparable between PFA-P (22.8%) and PFA-F (17.6%), with a hazard ratio of 0.83 (95% CI 0.36–1.95; *p* = 0.673) (Fig. 1).

### 3.3 Periprocedural characteristics

No significant difference were observed between the PFA-P and PFA-F groups regarding skin-to-skin time (45.0 [21.8] vs. 44.5 [22.0] min, *p* = 0.356), radiation dose (12.1 [13.7] vs. 11.0 [9.1] Gy*cm^2^, *p* = 0.304) or amount of contrast medium between the groups (50.0 [20.0] vs. 45.0 [15.0] ml, *p* = 0.055), for PFA-P and PFA-F respectively. Fluoroscopy time (14.3 [4.2] vs. 11.9 [7.9] min, *p* = 0.009) as well as LA-dwell time (18 [6] vs. 14 [6] min, *p* < 0.001) were significantly longer for PFA-P compared to PFA-F (Table 2).

**Table 2:** Comparison of periprocedural parameter (skin-to-skin time, fluoroscopy time, LA-dwell time, radiation dose, propofol dose and fentanyl dose) between PFA-P and PFA—F.

Variable	PFA-P (n = 75)	PFA-F (n = 75)	p-value
Skin to skin time, median (IQR), min	45 (21.8)	44.5 (22.0)	0.356
Fluoroscopy time, median (IQR), min	14.3 (4.2)	11.9 (7.9)	**0.009**
LA-dwell time, median (IQR), min	18 (6)	14 (6)	**<0.001**
Radiation dose, median (IQR), Gy*cm^2^	12.1 (13.7)	11.0 (9.1)	0.304
Contrast medium, median (IQR), ml	50 (20)	45 (15)	0.055
Propofol dose, median (IQR), mg	300 (125)	295 (103)	0.523
Fentanyl dose, median (IQR), mg	0.1 (0)	0.1 (0)	0.316

PFA-P, Pulseselect™; PFA—F, Farapulse™; LA, left atrium; Gy, gray; IQR, interquartile range; SD, standard deviation.

The administered dose of propofol (300 vs. 295 mg, *p* = 0.523) and fentanyl (0.1 vs. 0.1 mg, *p* = 0.316) were comparable between the two groups (Table 2).

### 3.4 Safety outcomes

The overall complication rate was identical between the two groups (4.0% vs. 5.3%) and consisted exclusively of minor complications. These included one case of pseudoaneurysma in the PFA-P and PFA-F group and one AV-fistula in the PFA-F group, all managed conservatively. Moreover, one episode of hypoxia due to aspiration during the procedure in both groups one severe vagal reaction with asystole in the PFA-F group, necessitating transient pacing during the procedure and temporary coronary spasm in the PFA-P group (**Suppl. Table 1**).

### 3.5 Biomarkers of cardiac and neural injury

**Fig. 2:**
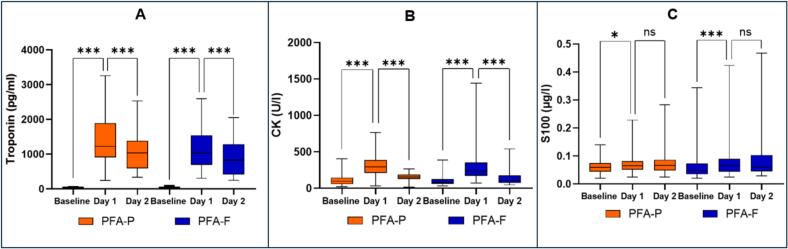
Temporal dynamics of A) Troponin, B) CK, C) S100 following PFA-P and PFA-F. Following PVI a significant increase of A) troponin, B) CK and C) S100 is observed in both PFA-P and PFA-F on first day after PVI compared to baseline. On the second day following the ablation A) troponin and B) CK decrease significantly in both groups, while no significant dynamic of C) S100 is observed in either PFA-P or PFA-F. ns, p > 0.05; *, p ≤ 0.05; **, p ≤ 0.01 ***, p ≤ 0.001.

Similar temporal dynamic change of troponin, CK and S100 was observed after both PFA-P and PFA—F, with a significant increase (*p* < 0.001) of both troponin and CK on day 1 compared to baseline (troponin: PFA—P: 1225 [950] vs. 9.5 [8.9] pg/ml; PFA—F: 1029 [817] vs. 11.0 [11.3] pg/ml) and CK (PFA—P: 292 [170] vs. 96.5 [85.8] U/l; PFA—F: 234 [173] vs. 84.5 [62.3] U/l), followed by a significant decrease on day 2 of both troponin (PFA—P: 1034 [762] pg/ml, *p* < 0.001; PFA—F: 829 [859] pg/ml, *p* = 0.001) and CK (PFA—P: 156 [57.8] U/l, p < 0.001; PFA—F: 106 [96] U/l, p < 0.001) compared to day 1 (Fig. 2).

A low but significant increase was observed in both groups on day 1 after PVI (PFA—P: 0.065 [0.03] vs. 0.06 [0.03] μg/ml, *p* = 0.015; PFA—F: 0.067 [0.04] vs. 0.048 [0.03] μg/ml, p < 0.001), while no significant changes were observed on the following day (Fig. 2C).

**Fig. 3:**
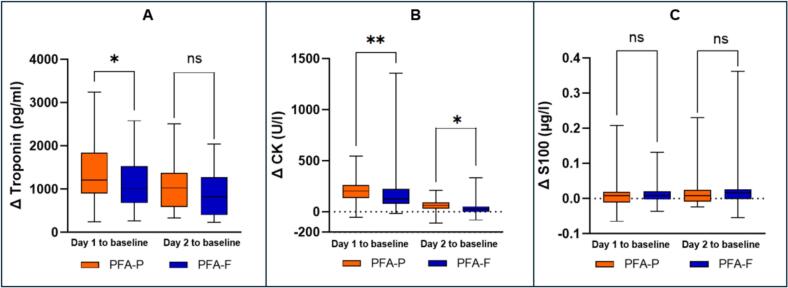
Comparison of A) Troponin, B) CK, C) S100 following PVI with PFA-P vs. PFA-F. Analysis of mean increment of serum levels post PVI revealed a significantly higher increase of A) troponin on day 1 compared to baseline and B) CK on both days compared to baseline following PVI with PFA-P compared to PFA-F. Levels of C) ΔS100 were comparably low in both PFA-P and PFA-F post ablation. ns, p>0.05; *, p≤ 0.05; **, p≤ 0.01.

Analysis of mean increment of serum levels post PVI revealed a significantly higher troponin elevation in the PFA-P group on day 1, (Δtroponin day 1: 1212 [930] vs. 1014 [809] pg/ml, for PFA-P and PFA-F respectively, *p* = 0.035), while on day 2 changes showed just a trend towards higher remaining troponin levels in the PFA-P group (1026 [758] vs. 821 [853] pg/ml, *p* = 0.061). Values of ΔCK were significantly higher in PFA-P compared to PFA-F patients both on day 1 (203 [118] U/l vs. 127 [141] U/l**,** *p* = 0.005) and day 2 (61 [58.3] vs. 26 [46.8] U/l**,** *p* = 0.011) for PFA-P and PFA- F patients respectively (Fig. 3). No significant differences were observed in ΔCK-MB levels (Fig. 3).

The extent of ΔS100 level changes were comparable between the groups (ΔS100 day 1: 0.008 [0.03] vs. 0.0075 [0.022] μg/l, for PFA-P and PFA-F respectively, *p* = 0.742; ΔS100 day 2: 0.007 [0.033] for PFA-P vs. 0.016 [0.026] μg/l for PFA—F, *p* = 0.291) (Fig. 3C).

### 3.6 Hemolysis parameters

**Fig. 4:**
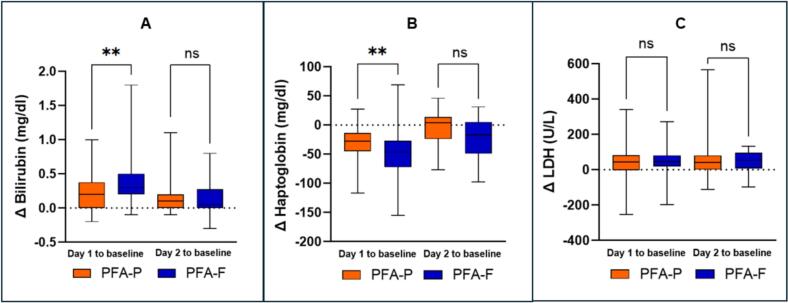
Comparison of A) Bilirubin, B) Haptoglobin, C) LDH following PVI with PFA-P vs. PFA-F. Analysis of mean increment of serum levels post PVI revealed a significantly higher increase of A) bilirubin and consecutive decrease of B) haptoglobin on day 1 compared to baseline after PFA-F compared to PFA-P. Levels of C) ΔLDH were comparable in both PFA-P and PFA-F post ablation. ns, p>0.05; *, p≤ 0.05.

Analysis of hemolysis parameters within both groups showed a significant elevation of bilirubin and LDH with a simultaneous decrease of haptoglobin in both groups on day 1 compared to baseline (**Suppl. Fig. 2**). Comparison between both groups revealed a significantly higher bilirubin increase on day 1 in the PFA-F vs. PFA-P group (Δ bilirubin day 1: 0.3 [0.3] vs. 0.2 [0.33] mg/dl, *p* = 0.004) in line with a concomitant significantly greater extent of haptoglobin decrease after PFA-F compared to PFA-P (Δ haptoglobin day 1: (−) 46.5 [44.8] vs. (−) 27.5 [30.5] mg/dl, *p* = 0.002). There were no significant differences between the two ablation systems observed in bilirubin level changes on day 2 (Δ bilirubin day 2: 0.1 vs. 0.1 mg/dl, for PFA-P and PFA-F respectively, *p* = 0.967), LDH levels on either day (Δ day 1: 44 vs. 46 U/l, *p* = 0.88 and Δ day 2: 41.5 vs. 54 U/l, for PFA-P and PFA-F respectively, *p* = 0.792) (Fig. 4). Hemoglobin (Hb) and creatinine dynamic was comparable between both groups, with no significant increase of creatinine on day 1 post ablation in both groups (**Suppl. Fig. 3**).

### 3.7 Learning curve

**Fig. 5:**
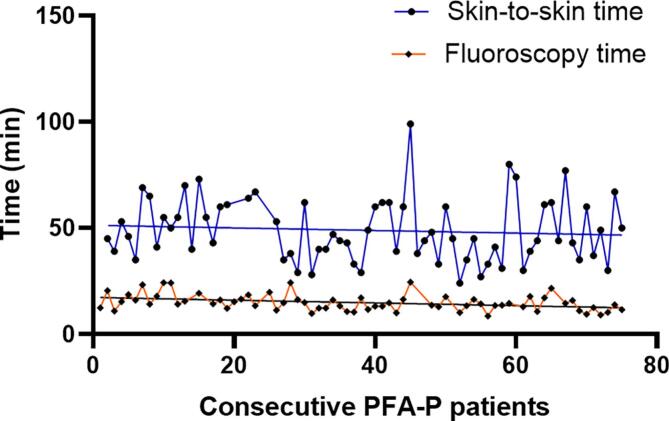
Trends in skin-to-skin time and fluoroscopy time in 75 consecutive PFA-P patients. Learning curve in consecutive PFA-P patient Analysis of skin to skin time and fluoroscopy time in 75 consecutive PFA-P patients shows a decreasing trend in both procedural outcomes over time.

A learning curve with a decreasing trend in skin-to-skin and fluoroscopy time is illustrated in Fig. 5. When comparing the first 25 PFA-P to the last 25 patients improvements in skin-to-skin time (55 vs. 44 min, *p* = 0.049), fluoroscopy time (16.1 vs. 13.5 min, *p* = 0.002), radiation dose (17.9 vs. 8.91 Gy*cm^2^, *p* = 0.029) and LA dwell time (21.3 vs. 18.2 min, *p* = 0.086) were observed, for the first and last 25 patients respectively (Table 3). Further, among the last 25 patients and with increased experience with PFA-P ablation, the operators opted for a direct TSP with FlexCath Countour sheath, eliminating the need for sheath exchange (Table 3).

**Table 3:** Learning curve- analysis of skin-to-skin time, fluoroscopy time, LA-dwell time, radiation dose and usage of FlexCath contour between the first and last 25 PFA-P patients.

Variable	First 25 PFA-P patients	Last 25 PFA-P patients	p-value
Skin to skin time, median (IQR), min	55 (19.8)	44 (25)	**0.049**
Fluoroscopy time, median (IQR), min	16.1 (4.9)	13.5 (3.8)	**0.002**
LA-dwell time, mean value ± SD, min	21.3 ± 4.94	18.2 ± 3.56	0.086
Radiation dose, mean value (SD), Gy*cm^2^	17.9 (17.3)	8.9 (10.4)	**0.029**
TSP performed with FlexCath Contour™, n (%)	2 (8)	20 (80)	**<0.001**

PFA-P, Pulseselect™; PFA—F, Farapulse™; LA, left atrium; Gy, gray; IQR, interquartile range; SD, standard deviation; TSP, transseptal puncture

## 4 Discussion

### 4.1 Efficacy and follow-up

In this contemporary patient cohort treated with the newly established PFA systems we demonstrated an overall high rate of procedural success and a very low complication rate with no observed differences among the two PFA technologies, which is in accordance with previous reports for PVI success rates with PFA-F [15]. Our study corroborates the results of previous reports on the acute efficacy of PFA-P in real world cohorts [11], [12], [16]. There was no significant difference in AT/AF recurrence rates between the two groups at 6-month follow-up. Although confirmation in larger cohorts with extended follow-up is warranted, our findings provide an initial perspective on recurrence rates after PVI using PFA-P in a direct comparison with PFA—F. This is particularly relevant as current real-world studies predominantly report acute procedural efficacy, with comparative long-term data between these two modalities still being lacking [11], [12], [16].

### 4.2 Safety

In our cohort, no major complications occurred, and there were no significant differences between the two groups concerning minor complications, all consistent with previous reports [8], [17]. This supports the feasibility of direct implementation of PFA-P in clinical practice, without raising concerns for the broader applicability, at least in centers which are familiar with single-shot PVI procedures.

### 4.3 Procedural outcomes

Both groups were comparable regarding skin-to-skin time and radiation dose. There were significantly longer LA dwell time and fluoroscopy time in the PFA-P group, which may be attributed to the frequent catheter repositioning in compliance with the manufacturer's instructions due to catheter designed gap [18]. However, analysis of the learning curve demonstrated a significant decrease in fluoroscopy time and a trend towards reduction in LA dwell time with increasing case load over time, highlighting the importance of operator experience with the device. Importantly, operators had extensive prior experience with PFA—F, whereas PFA-P was implemented during the study period, and thus differences in procedural parameters may in part reflect the early adoption phase and associated learning curve rather than differences attributable to the underlying technology itself. Prior real-world studies similarly reported no significant differences in procedure time between the two PFA modalities [11], [16]. However, a multicenter study by Abeln et al. demonstrated significantly longer procedure times for PFA—P, which might be attributed to the more frequent use of electroanatomical mapping in the PFA-P group, as well as the limited PFA experience of several participating U.S. centers [12], highlighting that operator experience with PFA is an important requirement for the efficient implementation of PFA—P.

In contrast to the existing approval studies for PFA-F [5] and the new PFA-P catheter [8], [16], our cohort demonstrated significantly shorter skin-to-skin time, LA dwell time, and fluoroscopy time. This could be attributed to the single-shot experience of the operators in our study, as well as the absence of the 20-min waiting period, which was implemented in prior studies before confirming complete PVI through entrance block [7], [18].

Another important safety consideration is that the operators opted to perform TSP directly with the FlexCath Contour™ sheath in 34 of 75 PFA-P ablations after initial experience with the system, thus avoiding the need for sheath exchange, and thereby potentially reducing the risk of air embolism.

Overall, these findings support the applicability of the new PFA technology in high volume, single-shot experienced facilities, without the need for extensive additional training.

### 4.4 Biomarkers of cardiomyocyte damage

Analysis of cardiac biomarkers revealed a higher troponin dynamic in the PFA-P group on day 2 and higher CK on both days post ablation. This may suggest a more pronounced myocardial cell damage following PVI with PFA-P compared to PFA—F. However, the clinical relevance of this finding remains uncertain, particularly as clinically meaningful outcomes were comparable between both groups. Previous studies reported a higher troponin release after PFA-F compared to thermal modalities [19], [20], [21], although a study from Kawamura et al. showed no difference in the extent of ablation lesions in 3-D Mapping following ablation with PFA and thermal modalities [22]. In addition, prior studies have shown that the influence of post-procedural troponin release on arrhythmia recurrence may differ between energy sources. While prior studies suggested an association between greater troponin release after RF and CBA ablation and lower AF recurrence [23], [24], the study by Popa et al., which showed evaluated biomarker release following both RFA and PFA, confirmed this influence on arrhythmia recurrence rate only for RFA, but not for PFA [25]. Together, these findings emphasize that the influence of biomarker release on arrhythmia recurrence rates may differ between thermal and non-thermal energy sources and raise important questions regarding the reliability and prognostic value of cardiac biomarker release following PFA. This pattern is also reflected in our cohort, where higher troponin release following PVI with PFA-P did not translate into higher short-term arrhythmia recurrence, as 6-month outcomes were comparable between PFA-P and PFA—F. However, these findings should not be interpreted as definitive evidence, as the relatively short follow-up duration precludes conclusions regarding long-term outcomes and lesion durability.

Larger studies with biomarkers analysis in combinations with information from serial 3-D Mapping and long-term follow-up data are needed to elucidate if the observed different dynamics of biomarkers between PFA-P and PFA-F persist and could help to predict durability of transmural lesions, freedom of AF recurrence or safety. In addition, profiling of biomarker dynamic post PFA-PVI might allow to separate post PFA-PVI patients presenting with chest pain without ST segment elevations in ECG from those with pericarditis or gastric pain to avoid unnecessary coronary angiography due to suspected acute coronary syndrome. Zeljkovic et al. demonstrated that higher post-ablation troponin release after RFA and CBA was associated with an increased rate of pericarditis [26]. Although the results cannot be directly extrapolated to our PFA cohort, they emphasize the clinical relevance of biomarker dynamics in the assessment of post-procedural chest pain.

### 4.5 Markers of neural injury

We report lower levels of S100 compared to the published work for thermal modalities [14], [27], [28] with a statistically significant increase of S100 on day 1 after ablation, aligning with the results of previous studies investigating the S100 dynamic post PVI in PFA-F [29]. We did not find difference in mean increments of S100 between PFA-P and PFA-F post ablation. These results are consistent with previous studies using PFA energy [27], [28], [30] and confirm the lack of relevant neural injury for PFA—P. However, it should be noted that, in contrast to the study by Tohoku et al., we did not perform post-procedural MRI to exclude silent cerebral ischemia as a potential contributor to S100 release [29]. As Tahoku et al. failed to show a clinical advantage of S100 as a surrogate marker for recurrence prediction following PFA-F [29] it is reasonable to expect that S100 levels have no predictive value for arrhythmia recurrence post PFA-P either. In view of all published studies, it seems that PVI by PFA does not result in significant neural injury compared to thermal ablation modalities [31], [32].

### 4.6 Hemolysis

PFA has been associated with significant hemolysis [8], [33], with a higher number of energy deliveries being linked to a greater hemolysis severity [33]. The results of our study point to a possibly higher extent of hemolysis following PFA-F compared to PFA—P, reflected by a significantly higher increase of bilirubin and a significant decrease of haptoglobin serum levels following ablation with PFA-F compared to PFA—P, while using a comparable number of energy deliveries. This observation is consistent with findings from a previous study investigating hemolysis in PFA-P and PFA-F in a smaller cohort [34], and our results extend and confirm these insights in a larger, directly comparative cohorts. Taking into consideration that a critical threshold of 1500 V/cm has been identified to achieve stable electroporation of human erythrocytes using 100 μs pulses [35] a possible reason for an increased level of hemolysis parameters in patients treated with PFA-F may be due to higher electrical field strength of PFA-F compared to PFA—P. While higher voltage output is used in PFA-F (2000 V) than PFA-P (1500 V), the exact electroporation parameters including strength of electrical field are propriety information of both Boston Scientific and Medtronic. Therefore, further studies including larger number of patients are warranted to confirm our results and elucidate if PFA-F may lead to more hemolysis than PFA—P. Of note, in none of the PVI patients clinically relevant hemolysis was observed.

### 4.7 Learning curve

Despite the relatively small sample size and in concurrence with the reported experiences of another high volume ablation center [11] we observed a significant learning curve with PFA—P, reflected in a significantly shorter skin-to-skin time, fluoroscopy time and a trend towards shorter LA dwell time within a short time period. Moreover, due to the comparably small sheath of 10 Fr. in PFA—P, operators opted to proceed the TSP with FlexCath Contour™ sheath directly, thereby increasing the safety in regard to air embolism that can occur during sheath exchange [36]. These findings show that PFA-P can be quickly, safely and effectively implemented in PFA-F experienced centers.

### 4.8 Limitations

Our study has several limitations that should be considered when interpreting the results. The follow-up duration in our study was limited to 6 months, which precludes any definitive conclusions regarding the long-term efficacy or the potential superiority of one PFA technology over the other. Larger studies with extended follow-up are needed to address these questions.

The relatively small cohort size limits the generalizability of our findings. While our results provide valuable insights, larger multicenter registries or studies are required to confirm these outcomes and to explore the broader applicability of the observed trends. Furthermore, the comparison between both PFA systems was performed under conditions of unequal procedural maturity, as operators had extensive prior experience with PFA—F, whereas PFA-P was introduced during the study period. Consequently, differences in procedural parameters may in part reflect the early adoption phase and associated learning curve rather than being solely attributable to the underlying technology.

This was a monocentric study conducted in a high-volume electrophysiology center. The results may not fully reflect the learning curve or procedural outcomes at less experienced or lower-volume institutions.

The standard PFA-based PV isolation protocol at our center does not routinely include waiting times post-ablation for confirmation of PV entrance block. This could influence the acute procedural success rates and the comparability to studies that utilize more comprehensive mapping or monitoring protocols.

By acknowledging these limitations, our study aims to provide a transparent framework for the interpretation of the results, while highlighting areas for future research and methodological refinement.

## 5 Conclusion

In conclusion, our study provides strong evidence supporting the efficacy and safety of the newly established PFA-P system for PVI, demonstrating high procedural success and low complication rates in a contemporary patient cohort. Furthermore, the comparable periprocedural data and comparable 6-month recurrence rates between both groups suggest that experienced centers can implement PFA-P effectively without the need for extensive additional training. Finally, our findings suggest a greater extent of myocardial injury following PFA-P compared to PFA-F and are consistent with previous studies consistently showing a lack of relevant bystander neural injury following PFA-F or PFA—P, although we detected a higher extent of hemolysis following PFA-F compared to PFA—P.

## CRediT authorship contribution statement

**Christian Gold:** Writing – original draft, Methodology, Formal analysis, Data curation, Conceptualization. **Anastasia Falagkari:** Writing – original draft, Formal analysis, Conceptualization. **Florian Post:** Writing – review & editing, Validation, Formal analysis. **Victoria Johnson:** Writing – review & editing. **Esther Roth:** Writing – review & editing. **Sinan Justin Kühn:** Writing – review & editing. **Dominik Linz:** Writing – review & editing, Supervision. **Dobromir Dobrev:** Writing – review & editing, Supervision. **Julia Erath-Honold:** Writing – review & editing. **Laura Rottner:** Writing – review & editing. **David M. Leistner:** Writing – review & editing, Supervision. **Jana Kupusovic:** Writing – review & editing, Writing – original draft, Formal analysis, Conceptualization. **Reza Wakili:** Writing – review & editing, Supervision, Conceptualization.

## Declaration of competing interest

CG reports of travel grants from Boston Scientific. AF reports of lecture fees from Abiomed and Medpoint GmbH, grants from Boston Scientific and was a fellow of a Biotronik Fellowship. VJ reports consultant fees, travel support and lecture fees from Abbott Medicall GmbH, Medtronic GmbH, Pfizer Pharma GmbH, BMS GmbH & Co. KGaA, Astra Zeneca GmbH, Daichi Sankyo GmbH, Bayer Vital GmbH, Boehringer Ingelheim Pharma GmbH & Co. KG, NovoNordisk PharmaGmbH, Sanofi Aventis GmbH, Biotronik and was a Fellow Boston Scientific heart rhythm fellowship program. JEH reports consultant fees, travel support and lecture fees from ZOLL Medical; travel grants from Bayer Vital, Boehringer Ingelheim, Boston Scientific, St. Jude Medical/Abbott, Novartis and Pfizer; and lecture fees from Alexion, Servier, Medtronic, Pfizer and Bayer Vital and was a fellow of the Boston Scientific heart rhythm fellowship program and the Biotronik Digital International Fellowship. LR received consultant fees and travel grants from Medtronic, Abbott, and KODEX-EPD (Phillips). DD received honoraria for educational lectures from Daichi Sankyo GmbH. On behalf of DL, Maastricht University received consultant fees and lecture honoraria from Bayer, Boston Scientific, CardioFocus, Johnson & Johnson, Medtronic, Novartis, ZOLL Medical. JK reports lecture fee from ZOLL Medical; travel grants from Biotronik. RW was supported by the German Centre for Cardiovascular Research (DZHK) Partner site Munich (DZHK; 81 × 1600203, 81 × 1600204, 81 × 2600216, 81 × 2600232, 81 × 2600234). RW received funding from the European Union's Horizon 2020 research and innovation program under grant agreement No. 633193″ [CATCH ME]. RW was funded by the Deutsche Forschungsgemeinschaft (DFG; German Research Foundation – DO637/23–1; Project number 394433254 to RW).

## Data Availability

The data underlying this study are available from the corresponding author upon reasonable request.
