# Supplementary material for: PulseSelect vs FARAPULSE pulsed field ablation: Comparative analysis of myocardial, neural-injury and hemolysis biomarkers and short-term outcomes
Source: Int J Cardiol Heart Vasc. 2026 Jun 18;65:101957. doi: 10.1016/j.ijcha.2026.101957 (PMC13310936; doi:10.1016/j.ijcha.2026.101957)
Supplement: Supplementary file 1 — Supplementary Material. [file mmc1.docx]

**Supplementary Figure 1.**

**
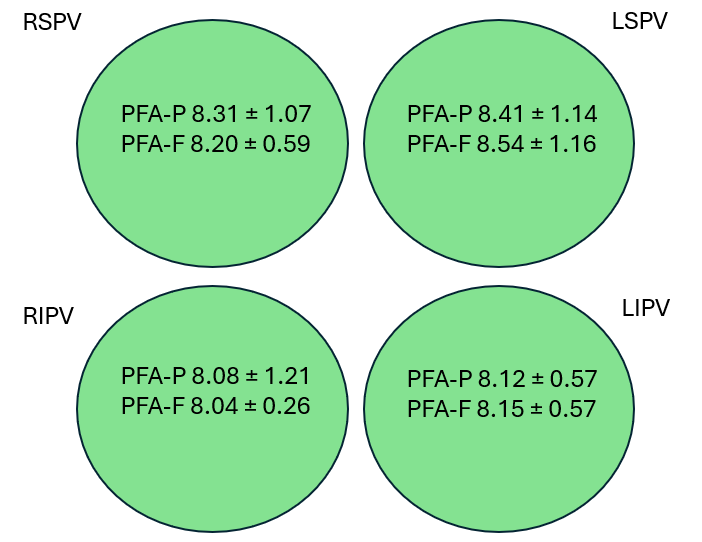
**

**Supplementary Figure 2.**


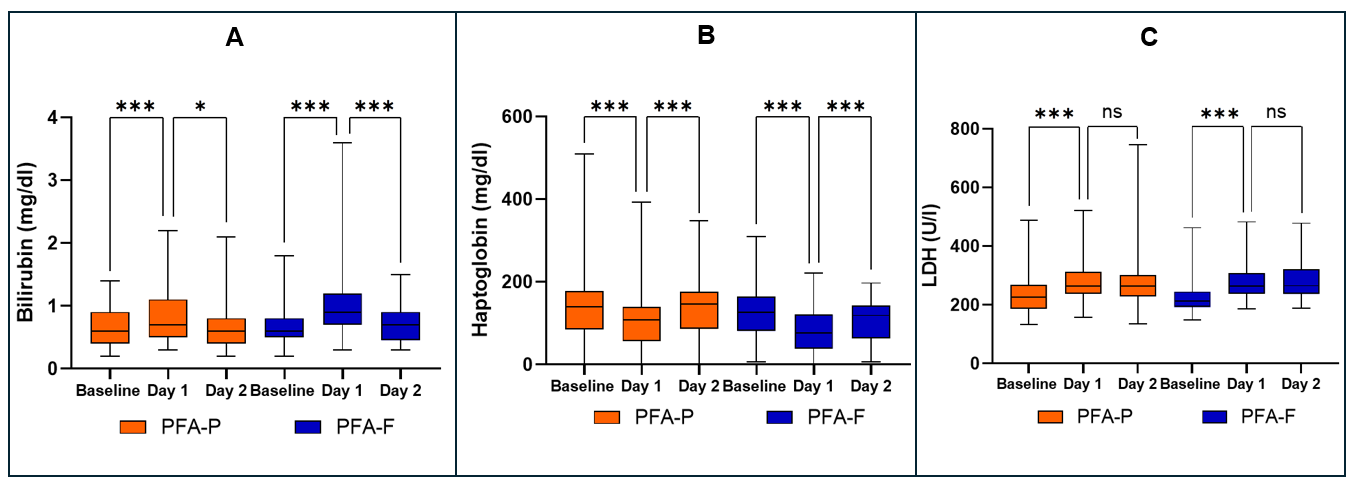


**Supplementary Figure 3.**


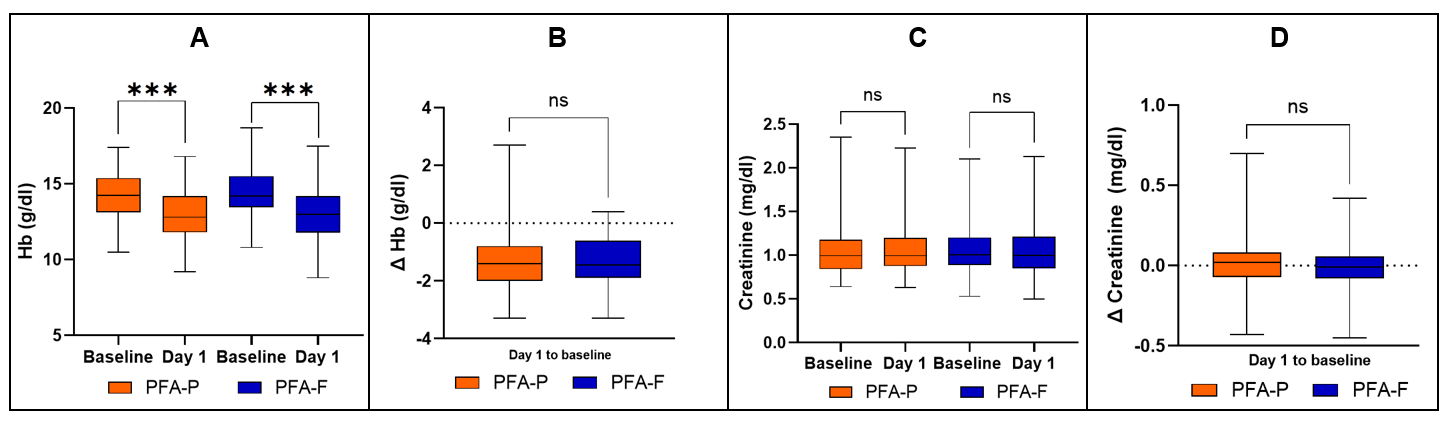


**Supplementary figure captions and figure legends:**

**Supplementary Figure 1.** Number of applications per vein

Comparison of mean applications per vein in PFA-P and PFA-F showed no significant difference between both groups for all pulmonary veins.

ns, p >0.05; RSPV, right superior vein; LSPV, left superior vein; RIPV, right inferior vein; LIPV, left inferior vein

**Supplementary Figure 2.** Temporal dynamics of A) Bilirubin, B) Haptoglobin, C) LDH following PVI with PFA-P and PFA-F

Following PVI a significant increase of **A)** bilirubin and **C)** LDH is observed in both PFA-P and PFA-F on the first day after PVI compared to baseline, with a consecutive significant decrease of **B)** haptoglobin. On the second day following the ablation **A)** bilirubin signficantly decreased in the PFA-F group while **B)** haptoglobin increased in both groups compared to day 1, while no significant dynamic of **C)** LDH is observed in either PFA-P or PFA-F. ns, p > 0.05; *, p ≤ 0.05; **, p ≤ 0.01 ***, p ≤ 0.001

**Supplementary Figure 3.** Comparison of A) Hemoglobin before and after PVI with PFA-P and PFA-F, B) $\Delta$Hemoglobin between PFA-P and PFA-F, C) Creatinine before and after PVI with PFA-P and PFA-F, D) $\Delta$Creatinine between PFA-P and PFA-F

Following PVI a significant decrease of hemoglobin is observed in both PFA-P and PFA-F groups on day 1 compared to Baseline (**A**), while the mean decrease of hemoglobin was comparable in both groups on day 1 compared to baseline (**B**). **C)** No significant dynamic of creatinine was observed either after PFA-P or PFA-F and **D)** mean increment of creatinine levels was comparable between both groups post PVI. Hb, Hemoglobin; ns, p >0.05; ***, p ≤ 0.001

**Supplementary Table 1. Comparison of complication rate between PFA-P and PFA-F**

| **Variable** | **PFA-P (n=75)** | **PFA-F (n=75)** | **p-value** |
| --- | --- | --- | --- |
| Overall complication rate, n (%)  *Pseudoaneurysma, n (%*)*  *Hypoxia due to aspiration, n (%*)*  *Vagal reaction with asystolie, n (%*)*  *AV-fistula, n (%*)*  *Temporary coronary spasm, n (%*)* | 3 (4)  *1 (33.3)*  *1 (33.3)*  *0 (0)*  *0 (0)*  *1 (33.3)* | 4 (5.3)  *1 (25)*  *1 (25)*  *1 (25)*  *1 (25)*  *0 (0)* | 1  *1*  *1*  *1*  *1*  *1* |

*% of total complications within group

PFA-P, Pulseselect™; PFA-F, Farapulse™; AV, arteriovenous
